# A Risk Evaluation Framework in System Control Subject to Sensor Degradation and Failure †

**DOI:** 10.3390/s24051550

**Published:** 2024-02-28

**Authors:** Tangxiao Yuan, Weilin Xu, Kondo Hloindo Adjallah, Huifen Wang, Linyan Liu, Junshan Xu

**Affiliations:** 1 School of Mechanical Engineering, Nanjing University of Science and Technology, Nanjing 210094, China; vleenxu@njust.edu.cn (W.X.); liulinyan@njust.edu.cn (L.L.); xujunshan@njust.edu.cn (J.X.); 2 LCOMS, University of Lorraine, 57070 Metz, France; kondo.adjallah@univ-lorraine.fr

**Keywords:** sensor failure, black box, risk prediction, risk assessment, decision-making, framework

## Abstract

Sensor degradation and failure often undermine users’ confidence in adopting a new data-driven decision-making model, especially in risk-sensitive scenarios. A risk assessment framework tailored to classification algorithms is introduced to evaluate the decision-making risks arising from sensor degradation and failures in such scenarios. The framework encompasses various steps, including on-site fault-free data collection, sensor failure data collection, fault data generation, simulated data-driven decision-making, risk identification, quantitative risk assessment, and risk prediction. Leveraging this risk assessment framework, users can evaluate the potential risks of decision errors under the current data collection status. Before model adoption, ranking risk sensitivity to sensor data provides a basis for optimizing data collection. During the use of decision algorithms, considering the expected lifespan of sensors enables the prediction of potential risks the system might face, offering comprehensive information for sensor maintenance. This method has been validated through a case study involving an access control.

## 1. Introduction

### 1.1. Dilemma of Real-Time Decision-Making

The rapid development of communication technology and big data processing has facilitated in-depth research on complex data-driven decision models in various industry fields, such as fault detection and diagnosis [1,2], real-time decision-making in power systems [3,4], etc.

These models are often categorized as black-box or gray-box models, with internal logic that is challenging to explain. In particular, when decision models are developed by third parties, users are unable to understand the specific operational mechanisms inside the box, leading to low interpretability and a lack of trust from users [5].

In practical applications, especially in scenarios involving real-time decision-making with immediate execution, big data-driven models face challenges in achieving effective implementation. For example, in the realm of real-time adjustment of process parameters, the absence of human intervention in the transition from decision-making to action execution poses a challenge for promptly evaluating the feasibility of decisions. This stands in contrast to fault detection and diagnosis, where decisions are not immediately executed by computers but necessitate human evaluation with subsequent action.

Furthermore, there is a distinction in data quality between the development and application phases of such algorithms [6]. Data cleaning is a critical step in constructing data-driven models, which often involves removing erroneous data and estimating missing information. However, in real decision-making scenarios in industry, decisions must be made regardless of how much information is missing from the input data. Therefore, data-driven models need to exhibit high robustness in dealing with missing information in input data.

In 2019, Holm [7] pointed out that when black-box methods can generate optimal results with low costs for errors, they can still provide value. However, it is crucial to ensure that algorithms possess confidence and low-risk characteristics before they are used in situations involving real-time decision-making and immediate execution. The algorithm under evaluation in this research maintains fixed inputs and outputs. Algorithms that do not adhere to fixed inputs and outputs are not addressed in the discussion.

### 1.2. Disadvantages of Algorithm Performance Indicators

Classification and pattern recognition algorithms can be assessed by several metrics, including accuracy, precision, recall, F1 score, receiver operating characteristic (ROC) curve, area under the ROC curve (AUC), confusion matrix, and mean average precision (MAP). These metrics collectively evaluate diverse facets of model performance, spanning classification accuracy, the model’s capability to correctly identify positive and negative samples, and its effectiveness across multiple categories. This comprehensive assessment provides an inclusive evaluation of algorithmic performance. Conversely, evaluation metrics for regression models primarily feature mean squared error (MSE), mean absolute error (MAE), and coefficient of determination (R^2^), among others. Table 1 summarizes these metrics.

While integral in assessing algorithmic performance from an algorithm-centric perspective, these evaluation metrics often lack consideration for real-world implications and user-centric factors. For instance, the confusion matrix provides probabilities of misclassifications, yet it falls short of delineating the tangible consequences of these misclassifications. The impact of misclassification, beyond statistical probabilities, results in risks that users are concerned about when wrong decisions are executed. Indeed, the consequences of misclassification include potential financial losses, equipment damage, and bodily injury risks, so the real-world implications need to be considered more broadly. Therefore, while these metrics offer valuable insights into algorithm performance, their interpretations must be complemented by a thorough understanding of the real-world ramifications of model errors on users and operational environments. Furthermore, the actual data and environment are often very different from the training data and laboratory environment. The evaluation indicators obtained by the algorithm in laboratory often make it difficult to convince users. The latter have nothing to worry about when executing algorithmic decisions in a low-risk environment. But when the algorithm is used in a high-risk environment, the degradation and failure of sensors and actuators can bias decision-making, thereby creating risks.

Many approaches can be used in control systems to tackle environmental changes and uncertainties. The robust optimization method accounts for system uncertainties and effectively counteracts system changes and interferences during the optimization process, thereby enhancing system robustness [8]. Additionally, fault diagnosis and fault-tolerant control promptly identify system faults or anomalies, implementing measures to uphold system stability. Fault-tolerant control systems are designed to handle potential system failures, ensuring continuous operation despite component faults [9]. In critical domains like aviation, nuclear power, and weaponry, where failures can pose significant risks, the importance of fault tolerance cannot be overstated. These methods aim to enhance the control system’s adaptability to fluctuations and uncertainties, increasing system stability, robustness, and reliability.

### 1.3. The Ignorance of Sensor Failure

Sensors are always at risk of failing in any real system, which results in inaccurate detection and potentially harmful execution. The sensor failures can be classified into four categories: bias, drift, performance degradation (loss of accuracy), and freezing [10].

Sensor failures can lead to high-risk consequences when using sensor data for decision-making. Therefore, it is crucial to consider potential failures and take preventive measures when relying on sensor data. This might include employing redundant sensor systems, regular sensor calibration, implementing fault detection and diagnostic algorithms, and establishing backup plans to address sensor failures. However, from a broader perspective of data collection systems, sensor failure detection alone cannot optimize the operation and maintenance of large-scale sensor networks. For instance, managing maintenance frequency under acceptable risks in extensive urban transit systems remains a crucial study area. Current periodic sensor replacement strategies result in the wastage of healthy sensors.

There is a scarcity of publicly available literature and datasets focusing on sensor failure. These failures may stem from hardware damage, misalignment, electromagnetic interference, or environmental changes. Manufacturer-provided lifespan estimates are often derived from accelerated tests or simulations in specific environments. However, actual operational conditions differ, making it challenging to predict sensor lifespans accurately. Hence, recording historical sensor failure data under real conditions is crucial. This information includes common failure types, vulnerable sensors, and anticipated failure frequencies within specific timeframes. Yet this information remains underappreciated in most scenarios. Accumulated failure data within control systems allows for the construction of statistical models for sensor lifespan and failure, aiding in predicting future failures.

Leveraging these data enables actions such as regular maintenance, component replacement, or optimizing sensor environments to extend lifespans and minimize future failures. Integrating historical statistical information assists designers and maintenance personnel in managing multi-sensor systems, enhancing reliability and stability.

### 1.4. Current Risk Evaluation Methods

Evaluating the consequence following an incorrect decision execution is integral to determining the acceptable level of accuracy reduction. In order to determine the “acceptable accuracy degradation”, we use the result of the quantitative risk assessment. The US Air Force [11] defined risk as the probability and severity of loss from exposure to a hazard. According to David Hillson [12], the risk is the result of one or more definite causes due to the occurrence of an uncertain event, which would lead to one or more effects on initial objectives. Markowski and Mannan [13] define risk as a combination of the severity of the consequences occurring in a certain accident scenario and its probability.

Assessment of risk often works as a management tool to assist decision-makers in identifying and addressing potential issues. Existing risk assessment methods include RPN (Risk Priority Number) for sorting internal system component failures and using fault trees [14], Bayesian networks [15], and Markov chains [16], which are used to study interrelationships among risks. Similarly, SPRC [17], FMEA [18], and influence diagrams [19] are adept at assessing the impact of singular events like floods or fires. These techniques provide avenues for quantitatively assessing continuous risks posed by sensor failures in information gathering, decision-making, and execution. However, due to the vague definition of risks and the diversity of risk indicators, scholars conduct limited systematic research, making it challenging to quantify risk assessments for model usage.

In this paper, risk is defined as the potential loss resulting from potential hazards. The risk value can be obtained by multiplying the probability of the hazard by the estimated loss, as proposed by Ni et al. [20], as expressed in Equation (1).

(1) Risk=(probability of hazard)×(estimated loss of damage),

where the value of probability of hazard and estimated loss of damage can be obtained based on historical data analysis or expert assessment.

### 1.5. Conclusions

With the advancement of communication technology and big data processing, many third-party and artificial intelligence decision models are being applied to system control. However, the inherent decision logic of these models is often inaccessible or challenging to explain to users. The above review indicates a relatively limited focus on the degradation and failure of sensors in system control. Additionally, there is a lack of research on the quantitative assessment of risks in system control.

In real-time scenarios, the data quality is often inferior to data cleaned for model training. In risk-sensitive situations, this impedes users’ confidence in these system control models. It is essential to delve deeper into the potential risks posed by data quality issues and provide additional risk information to empower users to utilize computer-generated decisions effectively.

This paper introduces a risk assessment framework to evaluate the risk associated with model misjudgments arising from sensor failures in the control system. By simulating failure scenarios and observing the output of the algorithm to these scenarios, the study investigates the resilience and reliability of the decision-making model. Furthermore, the paper explores using historical data, expert knowledge, or industry standards to identify sensor failure patterns and formulates statistical models for sensor failure lifespans in on-site environments. Leveraging this statistical model, the paper predicts the potential risks the system might face due to sensor failures. Finally, the effectiveness of the proposed method is validated in a case study involving subway access gate control scenarios.

## 2. Methods

In contrast to traditional model testing, which primarily focuses on functional testing, scenarios that involve high requirements for personal safety and equipment facility security necessitate a deeper consideration of the risks associated with sensor data quality. The scenario analyzed in this paper involves an application utilizing pattern recognition or classification algorithms.

### 2.1. Notations

Let us consider the following parameters in Table 2.

### 2.2. Evaluation Process

**Figure 1:**
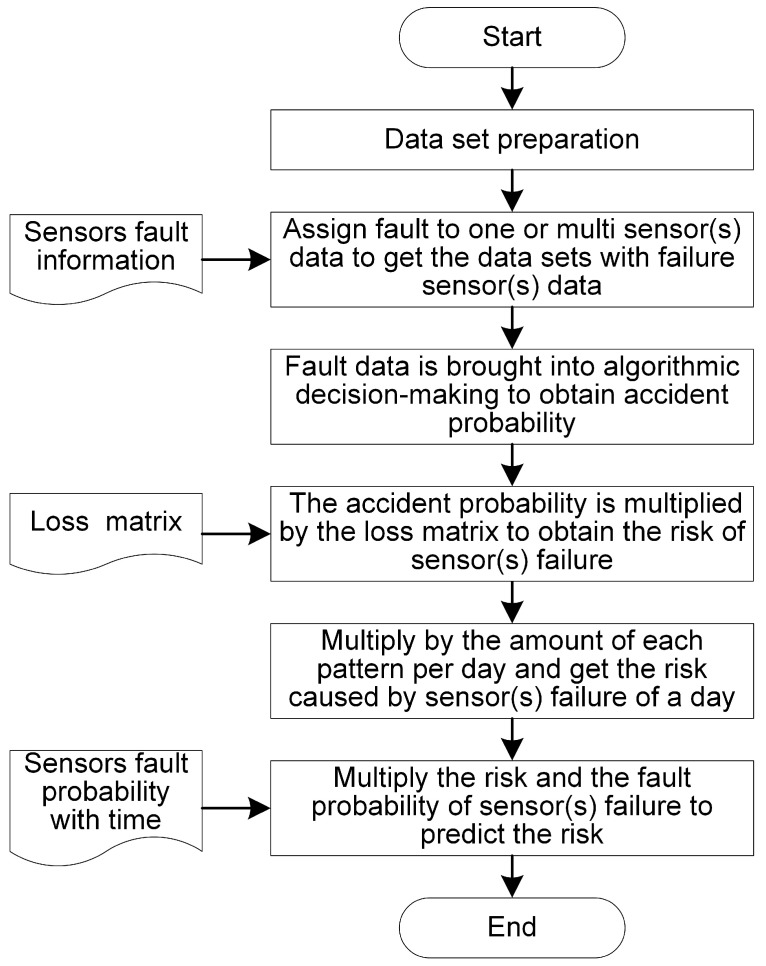
The specific sub-steps involved in simulating and predicting these details.

To evaluate the risks caused by sensor failures, this article adopts the following steps to analyze and address these issues. The detailed simulation process is shown in Figure 1.

- Step 1. Data collection: Collect data from the scenario, including sensor data and results.
- Step 2. Sensor failures identification and prediction: Identify the sensor failures, measurement accuracy, failure rate, etc.
- Step 3. Risk identification: Identify potential risks associated with sensor failures based on experience and expertise.
- Step 4. Risk quantification: Quantify potential losses resulting from identified risks based on experience and expertise.
- Step 5. Representation of the relationship between decision risks and data errors: Establish a mathematical relationship between decision risks and data errors.
- Step 6. Simulation and prediction.

The data collected in Step 1 serve as the inputs and outputs for the decision-making algorithm when there is no sensor degradation or failure. The result labels for these patterns are already associated with the function input before employing this method. Furthermore, the proportion of various patterns in the sample should also mirror real-world proportions. If the proportion of each pattern in the data set deviates from the actual scene, the correct proportion of each pattern needs to be counted separately.

In Step 2, a critical substep is constructing a data set for simulating decision-making with sensor faults by analyzing the data characteristics of the sensor under degradation and fault conditions.

Step 3 and Step 4 identify and evaluate the risks of executing incorrect decisions. The challenge lies in identifying risks the system may encounter based on experience, historical data, logs, and other information and quantitatively assessing the associated losses. It is emphasized that the risks discussed here arise from executing incorrect decisions and do not encompass losses resulting from actuator failures or human operational errors; these are beyond the scope of decision-related risks.

### 2.3. Scenario Description and Risk Evaluation

In this scenario, *n* sensor detection values are utilized as inputs for a decision-making algorithm. The sensors are indexed from 0 to *n*, each with a distinct failure type. The pattern recognition function can identify *k* patterns within the observed scene. Over a specified time frame involving *N* decision-making executions, the occurrence proportions of these *k* patterns are denoted as $a_{1}$ through $a_{k}$, as shown in Equation (2).

(2) $$P_{\mathrm{pattern}}={\left[\begin{array}{cccc} a_{1}, & a_{2}, & \cdots & a_{k} \end{array}\right]}_{1\times k}$$

Given that the system encounters *m* events, the loss matrix is derived from events resulting from erroneous recognition outcomes. The matrix’s diagonal values are set to 0, representing no loss when the correct action is taken. Equation (3) presents the loss matrix for the jth event.

(3) $$L_{j}={\left[\begin{array}{ccc} 0 & \cdots & l_{1k}^{j}\\ \vdots & \ddots & \vdots \\ l_{k1}^{j} & \cdots & 0 \end{array}\right]}_{k\times k}$$

where $l_{1k}^{i}$ is the value of loss when pattern 1 is recognized as pattern *k*.

When the ith sensor fails, the confusion matrix of the pattern recognition is $C_{i}$, which is shown as Equation (4).

(4) $$C_{i}={\left[\begin{array}{ccc} p_{11}^{i} & \cdots & p_{1k}^{i}\\ \vdots & \ddots & \vdots \\ p_{k1}^{i} & \cdots & p_{kk}^{i} \end{array}\right]}_{k\times k},$$

When the ith sensor fails, the specific risk matrix for the jth pattern, denoted as $r_{j}^{i}$, is described by Equation (5). Combining these individual risk matrices where the unit of risk is consistent yields the total risk matrix $r^{i}$ for each pattern in the event of the ith sensor’s failure over the given time span (as depicted in Equation (6)). The aggregated risk value $R^{i}$ is obtained by summing the elements within the matrix $r^{i}$, as outlined in Equation (7).

(5) $$r_{j}^{i}=N\cdot P_{\mathrm{pattern}}\cdot C_{i}\cdot L_{j},$$

(6) $$r^{i}=N\cdot P_{\mathrm{pattern}}\cdot \sum_{j=1}^{m}C_{i}\cdot L_{j},$$

(7) $$R^{i}=\sum_{p=1}^{k}\sum_{q=1}^{k}r_{pq}^{i},$$

By analyzing all sensor failures and sorting the obtained $R_{i}^{j}$, the impact of each sensor on the specific risk jth of the decision-making model can be ranked. If the risk is additive, sorting $R^{i}$ can obtain the ranking of potential threats to the safe operation of the system caused by each sensor.

If there is more than one type of sensor failure, the failures of the sensors can be analyzed separately according to the above process. For example, if sensor *u* and sensor *v* both fail, the risk can be obtained as Equations (8)–(10).

(8) $$r_{j}^{u,v}=N\cdot P_{\mathrm{pattern}}\cdot C_{u,v}\cdot L_{j},$$

(9) $$r^{u,v}=N\cdot P_{\mathrm{pattern}}\cdot \sum_{j=1}^{m}C_{u,v}\cdot L_{j},$$

(10) $$R^{u,v}=\sum_{p=1}^{k}\sum_{q=1}^{k}r_{pq}^{u,v},$$

### 2.4. Risk Prediction

The expected lifespan of sensors is closely tied to their operating environment. By recording sensor failure data in the control system and obtaining statistical information on sensor failures after a certain period of operation, the predicted risks faced by the system can be assessed.

Suppose the expected failure probability at time *t* for any sensor ith in the system is denoted as $p_{i}\left(t\right)$, assuming that sensor failures are independent events.

Therefore, the risk faced by the system due to a single sensor failure at time *t* can be derived using Equation (11):

(11) $$R=\sum_{i=1}^{n}\left(p_{i}\left(t\right)\left(\prod_{j=1}^{n}\left(1-p_{j}\left(t\right)\right)\right)R^{i}\right),i\neq j,$$

If any two sensors fail simultaneously, the system’s risk can be determined using Equation (12):

(12) $$R=\sum_{u=1}^{n}\sum_{v=1}^{n}\left(p_{u}\left(t\right)p_{v}\left(t\right)\left(\prod_{j=1}^{n}\left(1-p_{j}\left(t\right)\right)\right)R^{u,v}\right),j\neq u,and,j\neq v,$$

Similarly, this can be extended to assess the risk faced by the system when three or more sensors fail simultaneously. The sum of all ∑R values yields the total risk the system faces.

## 3. Case Study

This assessment framework evaluates decision-making risks caused by sensor degradation and failure. The above framework can be applied in scenarios using pattern recognition and classification algorithms. The main challenges of this framework lie in data acquisition and loss evaluation, both of which necessitate prolonged monitoring of the system to gather sufficient historical data. In the following subway access gate case, sufficient data have been collected in long-term subway operations. We can obtain risk-related event probability, loss, sensor life expectancy, and other information from historical operation data and expert knowledge.

In this case, the algorithm of the access gate is a pattern recognition model based on a two-dimensional interpolation algorithm and convolution neural network. The inputs for this decision model are the status matrices of 11 photoelectric sensors in the entrance detection area. The status matrix is the status of the 11 sensors over a period of time after the passenger enters the detection area and before the passenger reaches the gate. The output is the opening time. The model can measure the walking speed of a passenger. If the passenger walks slowly, the opening time will be longer. The algorithm is based on a two-dimensional interpolation algorithm and convolution neural network.

According to the information from Nanjing Metro Ltd., Nanjing, China, millions of passengers take the metro daily in Nanjing. At the busiest subway station, each turnstile has an average daily traffic of more than 10,000 passengers. The safety of subway gate control is directly related to passenger travel experience and safety. For example, on 29 June 2012, a child, accompanied by a senior passenger, was injured when the subway gate suddenly closed, causing abdominal injuries and intestinal perforation. This algorithm is designed with the assumption that the sensor data are reliable and of good quality. When a sensor fails, the algorithm’s performance may be adversely affected. In such situations, the reliability and accuracy of the algorithm’s judgments can be compromised.

### 3.1. Scenario Description

**Figure 2:**
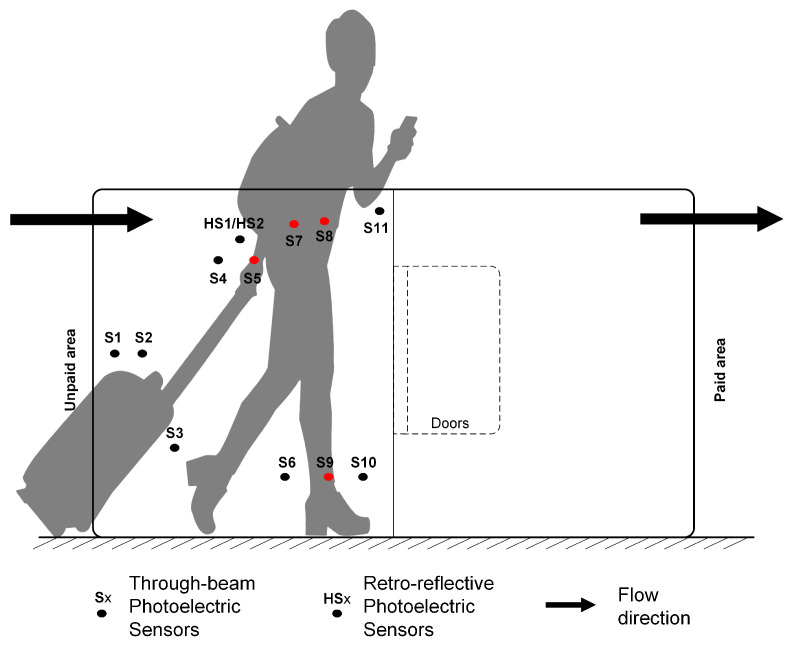
The positions of sensors [22].

The layout of the sensors shown in Figure 2 is designed based on the human body line graph model established by J.-H. Yoo et al., according to the knowledge of human anatomy that allows us to recognize the human gait [21].

Assumed that the distribution of daily pedestrian traffic follows a normal distribution with a mean of 10,000, and 99% of the pedestrian traffic falls within a two-sided confidence interval of 8500 to 11,500. There are seven patterns of passage for adults. The pattern and assumed proportion of each pattern in the given station are introduced in Table 3. The opening time of the turnstile depends on the recognition of pedestrian patterns and walking speed.

### 3.2. Sensor Fault Identification

**Figure 3:**
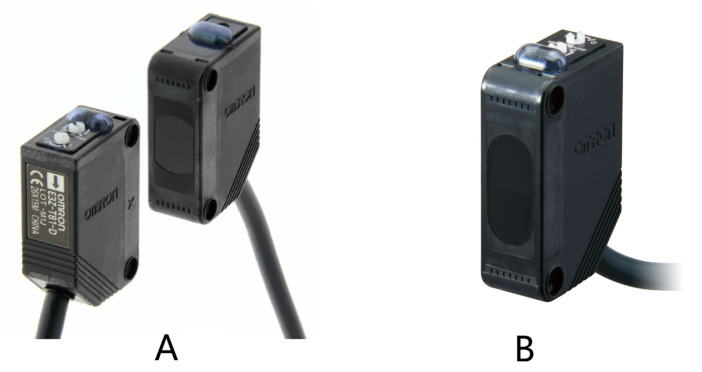
Photoelectric sensors ((**A**) through-beam sensor; (**B**) retro-reflective sensor) [22].

Omron E3Z-T61 and E32G-D62 photoelectric are the sensors used in the access gate, as shown in Figure 3. The setting of the sensor is low level (output with 0) when it is unblocked and high level (output with 1) when it is blocked.

There are two types of photoelectric sensor failure. The first failure mode is mode 0. Regardless of an obstruction, the sensor always outputs a signal with 0. The second failure mode is mode 1: the sensor always outputs a signal with one. Besides the above two sensor failures, assume that:

- The detection accuracy of sensors is 100%, and there are no obstructions in the environment other than pedestrians.
- Apart from the above two failure modes, the sensor does not have any other failure modes

### 3.3. Risk Identification and Quantification

Misjudgment of pattern recognition will result in two risks for the subway operator. One risk is the ticket loss (TL). For example, the behavior of tailgating into the station is not identified, resulting in ticket loss. According to Equation (3), when the pedestrian pattern recognition is wrong, the ticket loss matrix (TL) is shown in Table 4.

Another risk is customer complaints (CCs) of the early closing of the door caused by a failure of judgment, which leads to people being trapped. The mechanical structure of the gate machine has a personal safety protection function, and it can bounce back when it touches people, so it rarely causes injuries. Some customers concerned about the gate’s early closing complained when the turnstile touched their body. According to Equation (3), Table 5 shows the statistical results of passenger complaints about misjudgments.

### 3.4. Risk Evaluation before Application

#### 3.4.1. Risk Evaluation When Single Sensor Failures

According to Equation (4), 22 confusion matrices can be obtained after the simulation of each sensor failure. For example, when sensor 3 fails to mode 0, the identification results are presented in Table 6. Taking the first row in Table 6 as an example, after experiencing a mode 0 fault with sensor 3, the accuracy of correctly identifying pattern A as pattern A is 98.5%. A total of 1.5% of pattern A is identified as pattern D. The other 21 confusion matrices of other sensor failures are not introduced in detail.

Table 7 is the risks per day when a sensor fails. Based on the information provided in Table 7, it is evident that mode 0 failure of sensors poses a significantly higher risk of ticket loss compared to mode 1 failure. Sensor 1’s failure has an exceptionally high impact on the risk when other failures’ impact on the risk is small. Due to the different units of measurement for two types of risks, the numerical values of risks cannot be directly added together.

The importance ranking of sensor failures could be crucial in optimizing data collection before using the model. For instance, when a failure in a particular sensor could lead to unacceptable losses, it is essential to ensure the reliability of the information provided by this sensor in the design of the data collection network. The optimization of data collection can be achieved by deploying redundant sensors and incorporating different types of sensors.

#### 3.4.2. Risk Evaluation When Multi Sensors Fail Together

Within a device maintenance cycle, the likelihood of multiple sensors failing simultaneously is typically much lower than that of a single sensor failing. However, this possibility still exists. For instance, when a batch of sensors reaches its expected lifespan, it is plausible for multiple sensor failures to occur within the window between two maintenance sessions. The combinations of multiple sensor failures present numerous possibilities. In the case of the mentioned sensors, there are $3^{11}-22-1$ = 177,124 potential combinations in which multiple sensors fail simultaneously. Furthermore, considering only instances where two sensors fail simultaneously, there are still $C_{11}^{2}\times 4=220$ possible combinations. Due to space constraints, we will discuss only a few scenarios involving two sensors failing simultaneously.

**Figure 4:**
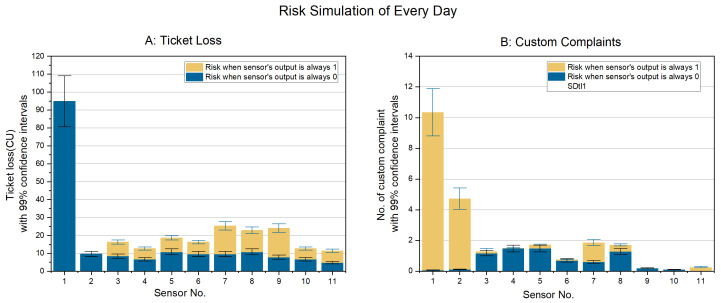
Simulation result of everyday ((**A**) risk of ticket loss (CU); (**B**) risk of customer complaints).

According to the risk simulation outcomes for individual sensor failures (refer to Figure 4), the most notable influence on customer complaint risk emerges when sensor 1 is combined with failure mode 1, and sensor 2 is combined with failure mode 1. Conversely, the highest risk concerning ticket losses arises from sensor 1 combined with failure mode 0. As a result, this study has opted to focus on simultaneous failure of sensors 1 and 2.

After the same process of risk simulation in Section 3.4.1, the risks under these two failure modes can be obtained, as shown in Table 8.

In Table 8, it is apparent that the combined failure to 0 of sensor 1 and sensor 2 concurrently leads to a substantial increase in the risk of ticket loss compared to the risks associated with individual sensor failures. However, the risk outcomes of other sensor failures did not change significantly.

### 3.5. Risk Evaluation during Application

#### 3.5.1. Sensor Fault Detection and Lifespan Distribution Model Construction

The system utilizes redundant information to discern whether each sensor is experiencing a fault, relying on mutual comparisons of signal variations among the sensors. This method assumes that most of the 11 sensors will not fail simultaneously. If other sensors detect someone passing through while a specific sensor shows no change in its signal level for ten consecutive instances, it can be conclusively determined that this sensor is faulty, and its current state output represents the fault mode. We can construct a database concerning sensor lifespans by embedding this sensor fault diagnosis within the control system, thereby establishing the expected lifespan distribution for such sensors.

**Figure 5:**
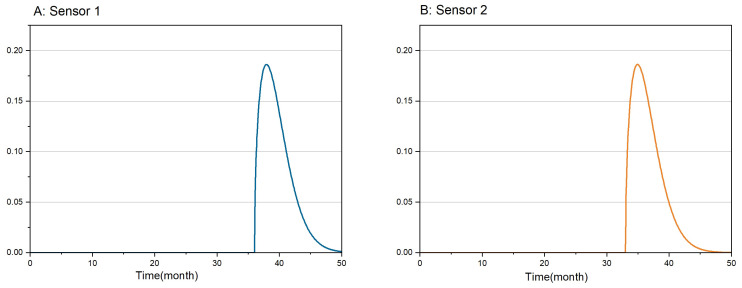
Sensor lifespan expectancy distribution density.

Assume that due to the different installation times of the two sensors, the expected life of the sensor follows a Weber distribution $f\left(t|c,\lambda \right)=\frac{t}{\lambda }{\left(\frac{c}{\lambda }\right)}^{c-1}e^{-{\left(t/\lambda \right)}^{c}}$, as shown in Equations (13) and (14). Assume that the sensors fail on the first day of the month.

(13) $$f_{1}\left(t\right)=\left\{\begin{array}{cc} 0 & 0\leq t\leq 36\\ \frac{1.5}{4}{\left(\frac{t-36}{4}\right)}^{0.5}e^{-{\left(\left(t-36\right)/4\right)}^{1.5}} & t>36 \end{array}\right.,$$

(14) $$f_{2}\left(t\right)=\left\{\begin{array}{cc} 0 & 0\leq t\leq 33\\ \frac{1.5}{4}{\left(\frac{t-33}{4}\right)}^{0.5}e^{-{\left(\left(t-33\right)/4\right)}^{1.5}} & t>33 \end{array}\right.,$$

where: c=1.5, λ=4, *t* is the sensor usage time (month). $f_{1}$ and $f_{2}$ represent the expected lifespan density functions of sensor 1 and sensor 2, respectively. The sensor’s lifespan expectancy distribution densities are shown in Figure 5.

#### 3.5.2. Risk Prediction

This section assumes that apart from sensor 1 and sensor 2, all other sensors have no faults. As indicated in Table 7 and Table 8, the risks associated with various types of faults and their combinations for sensors 1 and 2 are analyzed in the system.

Let us assume the sensor fails at the start of each month and the maintenance team corrects it once a month. With a month typically having 30 days, this would imply the sensor experiences a failure every 30 days and is corrected at the end of each 30-day period.

The failures of different sensors are independent events, and the probability of both sensors failing simultaneously is the product of their individual failure probabilities. According to Equations (8)–(12), and Table 7 and Table 8, the function of the risk of ticket loss and custom complaints per month is shown in Equations (15)–(20).

(15) $$\begin{array}{cc} R_{\mathrm{TL}}\left(t\right) & =R_{\mathrm{TL},1}\left(t\right)+R_{\mathrm{TL},2}\left(t\right)+R_{\mathrm{TL},1\&2}\left(t\right), \end{array}$$

where

$R_{\mathrm{TL},1}\left(t\right)$ is the ticket loss at time *t* when only sensor 1 fails.

$R_{\mathrm{TL},2}\left(t\right)$ is the ticket loss at time *t* when only sensor 2 fails.

$R_{\mathrm{TL},1\&2}\left(t\right)$ is the ticket loss at time *t* when sensor 1 and sensor 2 both fail.

(16) $$\begin{array}{c} R_{\mathrm{TL},1}\left(t\right)=\frac{1}{2}\times \left(94.8+0\right)\times 30\times \int_{0}^{t}f_{1}\left(u\right)du\left(1-\int_{0}^{t}f_{2}\left(v\right)dv\right), \end{array}$$

(17) $$\begin{array}{cc} R_{\mathrm{TL},2}\left(t\right) & =\frac{1}{2}\times \left(9.6+0\right)\times 30\times \int_{0}^{t}f_{2}\left(v\right)dv\left(1-\int_{0}^{t}f_{1}\left(u\right)du\right), \end{array}$$

(18) $$\begin{array}{c} R_{\mathrm{TL},1\&2}\left(t\right)=\frac{1}{4}\times \left(336.13+10.99+0+0\right)\times 30\times \int_{0}^{t}f_{1}\left(u\right)du\int_{0}^{t}f_{2}\left(v\right)dv. \end{array}$$

The $R_{\mathrm{TL}}\left(t\right)$ can be simplified to Equation (19).

(19) $$\begin{array}{c} R_{\mathrm{TL}}\left(t\right)=1037.4\int_{0}^{t}f_{1}\left(u\right)du\int_{0}^{t}f_{2}\left(v\right)dv+1422\int_{0}^{t}f_{1}\left(u\right)du+144\int_{0}^{t}f_{2}\left(v\right)dv, \end{array}$$

where:

$\int_{0}^{t}f_{1}\left(u\right)du$ is the probability of sensor 1 failing at time *t*.

$\int_{0}^{t}f_{2}\left(v\right)dv$ is the probability of sensor 2 failing at time *t*.

$\frac{1}{2}\times \left(94.8+0\right)\times 30$ is the average ticket loss risk per month when sensor 1 fails (2 failure modes) according to Table 7.

$\frac{1}{2}\times \left(9.6+0\right)\times 30$ is the average ticket loss risk per month when only sensor 2 fails (2 failure modes) according to Table 7.

$\frac{1}{4}\times \left(336.13+10.99+0+0\right)\times 30$ is the average ticket loss risk per month when sensor 1 and sensor 2 both fail according to Table 8, with four failure mode combinations with equal probability.

Similarly, the risk of custom complaints in the future can be obtained as Equation (20).

(20) $$\begin{array}{cc} R_{\mathrm{CCs}}\left(t\right) & =R_{\mathrm{CCs},1}\left(t\right)+R_{\mathrm{CCs},2}\left(t\right)+R_{\mathrm{CCs},1\&2}\left(t\right)\\ \; & =-39.225\int_{0}^{t}f_{1}\left(u\right)du\int_{0}^{t}f_{2}\left(v\right)dv+155.25\int_{0}^{t}f_{1}\left(u\right)du+70.8\int_{0}^{t}f_{2}\left(v\right)dv, \end{array}$$

where

$R_{\mathrm{CCs},1}\left(t\right)$ represents the customer complaints at time *t* when only sensor 1 fails.

$R_{\mathrm{CCs},2}\left(t\right)$ represents the customer complaints at time *t* when only sensor 2 fails.

$R_{\mathrm{CCs},1\&2}\left(t\right)$ represents the customer complaints at time *t* when sensor 1 and sensor 2 both fail.

**Figure 6:**
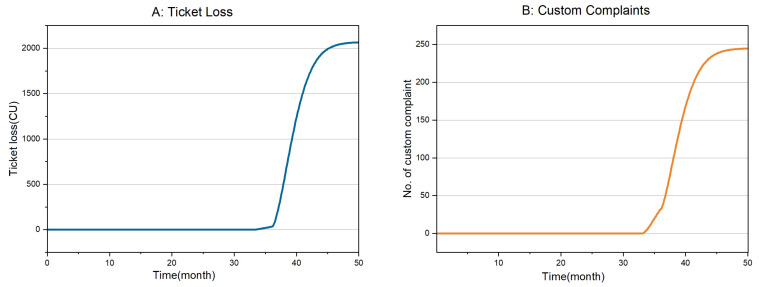
Risk prediction with time.

After the simulation, Figure 6 can be obtained. Both expected risks increase with time. Neither line is a smooth curve, and the turning point around 36 months is due to both sensors’ increased risk of simultaneous failure.

Based on risk prediction results, risk-based maintenance strategies can be developed. A risk-based maintenance strategy is a macro-maintenance strategy, which considers possible risks in the environment caused by failure. Some sensor degradation or failure will not cause significant losses, far from reaching the risk threshold. Then, the system can wait until the risk comes to the threshold before maintenance.

## 4. Discussion

The above case analysis can help users more clearly measure the risks caused by this decision-making algorithm in access gates. From Figure 4, we can determine the risk caused by the failure of each sensor on the day under the current sensor arrangement and the current passenger flow situation. It can also be understood that sensors 1 and 2 are susceptible to the risk of customer complaints. The risk of ticket loss is sensitive to the mode 0 failure of sensor 1. When the subway company plans to optimize the data collection, placing redundant sensors at essential locations to obtain information to reduce risks further will be a possible choice.

When predicting future risks, it can be seen from Figure 6 that when two sensors fail simultaneously, the risk rises more rapidly than when one sensor fails. After the subway company determines the maximum risk that the system can tolerate, it can select the maintenance time of the system according to Figure 6. This access gate decision model was developed by ourselves. The framework proposed played an essential role in convincing the subway company to use this decision model. However, no sensor failure has occurred since the access gate deployed is new.

The main challenges of this framework are data acquisition and loss evaluation. Using this method requires relying on the long-term historical operating data of the scenario to build a data set, which also includes historical sensor failure data in the scenario. In addition, quantitative loss assessment requires the intervention of domain experts.

Furthermore, the framework proposed in the article is to evaluate the risk of using pattern recognition algorithms. When the output of the decision-making algorithm is a continuous value, the specific evaluation process needs to be restructured. Although the idea of the framework still applies. For example, in real-time parameter adjustment in manufacturing, an additional probability function must be constructed to describe the relationship between the range of parameter over-adjustment and the resulting risk.

## 5. Conclusions

This article focuses on the impact of sensor failures on information processing and proposes a framework for evaluating decision risks related to sensor malfunctions. The assessment process involves six steps: data collection, sensor fault identification, risk identification, risk quantification, establishing the relationship between decision risk and data quality functions, and simulation and evaluation. The strengths of this evaluation framework lie in its comprehensive coverage of various aspects of the system, including information acquisition, decision-making, execution, and impact. The method overcomes the limitations of existing assessment metrics and simplistic functional tests. From a quantitative perspective, the evaluation framework presented in this paper provides decision-makers with more precise risk information.

The applicability of this assessment framework is not confined to specific case studies but can be extended to various fields and industries. In manufacturing, particularly in scenarios involving complex production processes and real-time decision-making, the framework can assess the potential impact of sensor failures on production quality and efficiency, such as real-time adjustment of process parameters. In the healthcare sector, the framework can be applied to analyze the quality of medical equipment data and evaluate the potential patient risks associated with equipment malfunctions or inaccurate data for medical decision-making. In the financial industry, the framework can assist in assessing financial decisions using machine learning algorithms, especially when considering market fluctuations and data uncertainties. In the energy sector, particularly in monitoring and controlling complex energy systems, the framework can be employed to assess the potential risks associated with sensor data quality and the energy supply chain. In summary, the principles and methods of this risk assessment framework can be generalized to many fields and industries that require real-time decision-making and are influenced by uncertain factors.

## Figures and Tables

**Table 1:** Performance evaluation metrics for models.

Metric	Description
Accuracy	Proportion of correctly classified samples.
Precision	Proportion of correctly predicted positive samples.
Recall	Proportion of actual positive samples correctly predicted.
F1 Score	Harmonic mean of precision and recall, balancing both metrics.
ROC and AUC	Graphical representation of true-positive rate against false-positive rate, with AUC measuring the model’s ranking ability.
Confusion Matrix	Matrix displays true positives, false positives, and false negatives.
MAP	Average precision across different classes is common in tasks like object detection.
MSE	Average of squared differences between predicted and actual values, measuring the size of deviations.
MAE	Average of absolute differences between predicted and actual values, indicating the average discrepancy.
R^2^	Measures the proportion of variability in the data explained by the model, ranging from 0 to 1, where closeness to 1 signifies better fit.

**Table 2:** Notion.

Notion	Explanation
$a_{i}$	The proportion of pattern *i*.
$p_{i}\left(t\right)$	The probability of sensor ith failing at time *t*.
$C_{i}$	The confusion matrix of the pattern recognition when the ith sensor fails.
$p_{jk}^{i}$	The percentage of pattern *j* recognized as pattern *k* when the ith sensor fails.
$L_{j}$	The loss matrix of the jth event
$l_{ik}^{j}$	The loss of the jth event when pattern *i* is recognized as pattern *k* when the jth sensor fails.
$r_{j}^{i}$	The matrix of the jth risk when the ith sensor fails.
$r^{i}$	The matrix of total risk when the ith sensor fails.
$R^{i}$	The total risk (value) when the ith sensor fails.
$R_{\mathrm{TL},i}\left(t\right)$	The ticket loss at time *t* when only sensor ith fails.

**Table 3:** Patterns and their proportion at the given station [22].

Patterns	Explanation	Proportion
Pattern A	One passer with backpack	0.4995
Pattern B	One passer pulling the suitcase	0.05
Pattern C	One passer pushing the suitcase	0.05
Pattern D	Only a person	0.39
Pattern E	Double person(tailgating)	0.01
Pattern F	One passer with wheelchair	0.0002
Pattern G	One passer with crutches	0.0003

**Table 4:** Ticket loss when pattern recognition is wrong (CU).

TL Matrix	A ^1^	B	C	D	E	F	G
Real A							
Real B							
Real C							
Real D							
Real E	6	6	6	6		6	6
Real F							
Real G							

^1^ Recognized as Pattern A.

**Table 5:** Number of passenger complaints when pattern recognition is wrong (every 100 times).

CCs Matrix	A ^1^	B	C	D	E	F	G
Real A							
Real B				2	2		
Real C				10	10		
Real D							
Real E							
Real F	10	10	10	10	10		
Real G	10	10	10	10	10		

^1^ Recognized as Pattern A.

**Table 6:** Pattern recognition confusion matrix with sensor 3 failure (mode 0).

Confusion Matrix	A	B	C	D	E	F	G
Real A	0.985	0.000	0.000	0.015	0.000	0.000	0.000
Real B	0.294	0.564	0.000	0.115	0.000	0.000	0.027
Real C	0.000	0.000	1.000	0.000	0.000	0.000	0.000
Real D	0.100	0.000	0.000	0.900	0.000	0.000	0.010
Real E	0.003	0.000	0.000	0.008	0.987	0.000	0.003
Real F	0.000	0.000	0.000	0.000	0.000	1.000	0.000
Real G	0.039	0.000	0.000	0.039	0.000	0.000	0.922

**Table 7:** Average risks per day when sensor fails.

Sensor	TL (0) ^1^	TL (1)	CCs (0)	CCs (1)
1	94.8	0	0.07	10.28
2	9.6	0	0.11	4.61
3	8.4	7.8	1.17	0.12
4	6.6	6	1.48	0.03
5	10.8	7.8	1.48	0.24
6	9.6	6.6	0.7	0.06
7	9.6	15.6	0.6	1.27
8	10.8	12	1.28	0.43
9	7.8	16.2	0.19	0
10	6.6	6	0.1	0
11	4.8	6.6	0	0.26

^1^ The ticket loss when the sensor fails with an output of ‘0’.

**Table 8:** The risk stemming from the simultaneous failure of sensors 1 and 2.

Risk	TL (CU)	CCs
S1 (0)–S2 (0) ^1^	336.13	0.18
S1 (0)–S2 (1)	10.99	4.20
S1 (1)–S2 (0)	0	10.27
S1 (1)–S2 (1)	0	10.26

^1^ Sensor 1 (S1) and sensor 2 (S2) both fail to output 0.

## Data Availability

Data are contained within the article.

## Abbreviations

The following abbreviations are used in this manuscript:

ROC	Receiver operating characteristic.
AUC	Area under the ROC curve.
MAP	Mean average precision.
MSE	Mean squared error.
MAE	Mean absolute error.
R^2^	Coefficient of determination.
SPRC	Source–pathway–receptor–consequence.
FMEA	Failure mode and effects analysis.
TL	Ticket loss.
CCs	Customer complaints.
